# Aquifer-differentiated process-guided GRU for multi-timescale groundwater-level prediction

**DOI:** 10.1016/j.isci.2026.117832

**Published:** 2026-10-09

**Authors:** Xiandong Xie, Baizhong Yan, Tian Wang, Chuanlong Han, Cong Wei, Yanliang Zhai, Jialong Lv, Mingrong Zhang

**Affiliations:** 1 Hebei GEO University, Shijiazhuang 050031, China; 2 Hebei Key Laboratory of Mountain Geological Environment, Chengde, China; 3 Hebei Province Collaborative Innovation Center for Sustainable Utilization of Water Resources and Optimization of Industrial Structure, Hebei GEO University, Shijiazhuang 050031, China

**Keywords:** groundwater-level prediction, process-guided, GRU, multi-timescale prediction, sustainable groundwater management

## Abstract

Groundwater-level prediction supports water-resource management in overexploited aquifers, yet conventional data-driven models often struggle to represent pumping disturbances and contrasting shallow- and deep-aquifer responses. Using groundwater levels, meteorological variables, and agricultural pumping data from four shallow and four deep wells in Guantao County, North China Plain, we developed an aquifer-differentiated process-guided gated recurrent unit model for daily and dekadal (10-day) prediction. Compared with a conventional gated recurrent unit model, the process-guided model reduced group-mean root-mean-square error by 7.2% and 6.1% for shallow and deep wells at the daily scale and by 4.0% and 2.1% at the dekadal scale, respectively. Deep-well performance depended on the pumping-response window, with 60-day averaged pumping providing a stable configuration. These incremental gains show that incorporating pumping disturbances and aquifer-specific response differences as process guidance can improve groundwater-level representation and provide a modeling approach for groundwater management in agricultural abstraction areas.

## Highlights

• Lumped hydrological process residuals guide GRU groundwater-level prediction
• Agricultural pumping is explicitly embedded as a process-guidance term
• Shallow and deep wells use aquifer-differentiated process guidance
• Deep-well prediction depends on the cumulative pumping-response window

## Graphical abstract

**Figure:**
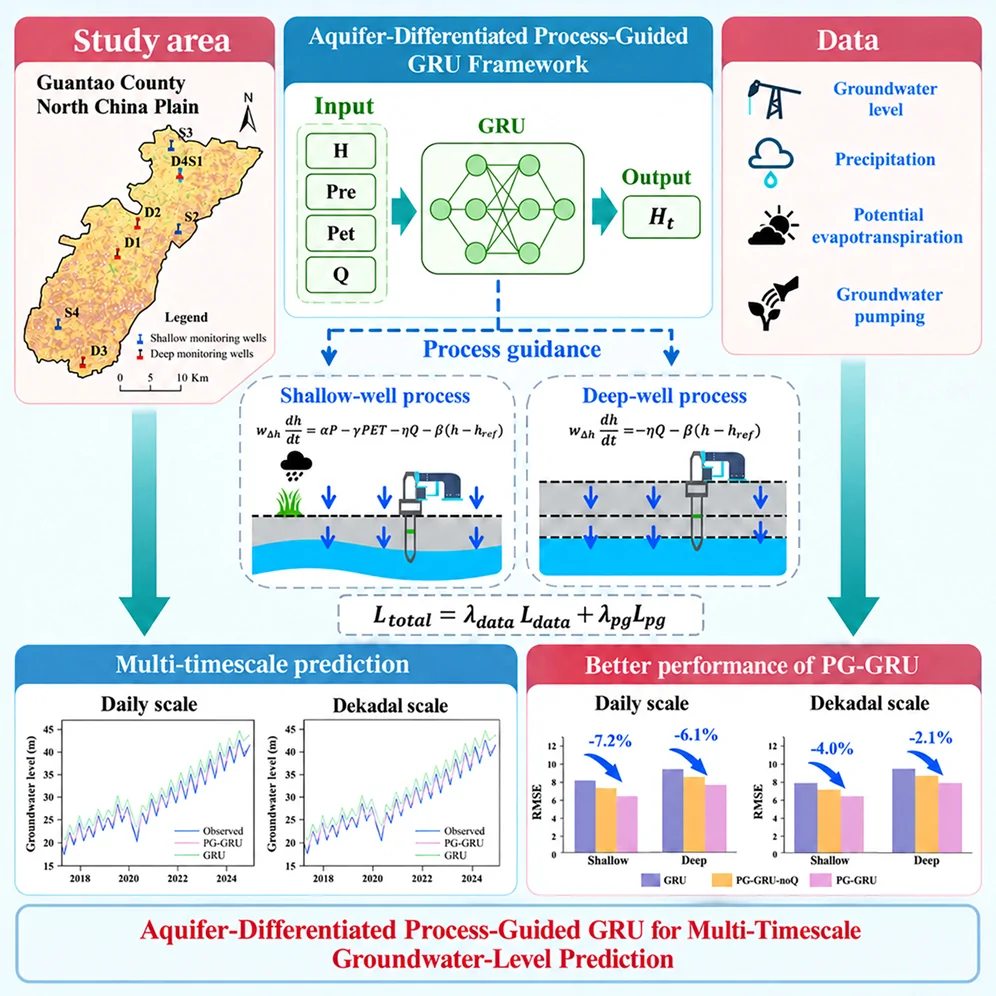

## Teaser

Environmental management; Environmental resource; Aquatic science

## Introduction

Groundwater is an essential water source for agricultural irrigation and regional water security. This is particularly the case in plain areas where surface water resources are relatively limited and cropping intensity is high. In such regions, groundwater has long played a critical role in sustaining agricultural production and ensuring food security. The North China Plain is a typical region in China with intensive agricultural groundwater abstraction. Although long-term irrigation pumping has supported agricultural development, it has also led to continuous groundwater-level decline, depletion of aquifer storage, and weakening of the regulatory capacity of groundwater systems. Under groundwater overexploitation, groundwater-level dynamics reflect not only natural processes such as precipitation infiltration and evapotranspiration, but also the persistent disturbance of agricultural pumping on groundwater systems.^1^ Therefore, accurate prediction of groundwater-level changes under agricultural pumping disturbances is essential for identifying groundwater system responses, optimizing irrigation pumping practices, and supporting sustainable groundwater resources management.

Groundwater-level variations are jointly affected by multiple factors, including meteorological conditions, irrigation pumping, aquifer structure, and the intensity of human activities, and are characterized by pronounced nonlinearity, lag effects, and multi-timescale variability.^2^ Conventional numerical groundwater models generally require relatively complete information on aquifer structure, boundary conditions, source-sink terms, and hydrogeological parameters. As a result, model construction is often complex, and parameter specification can be difficult. When pumping activities show strong spatiotemporal variability, monitoring data are limited, or aquifer systems are highly heterogeneous, the broader application of numerical models is often constrained.^3^^,^^4^^,^^5^^,^^6^^,^^7^^,^^8^ In contrast, data-driven models can automatically learn groundwater-level variation patterns from historical monitoring data and have strong advantages in handling nonlinear time-series problems. Consequently, they have been widely applied in groundwater-level prediction in recent years.

With the development of machine learning and deep learning methods, models such as artificial neural networks, support vector machines, random forests, extreme gradient boosting, long short-term memory networks, and gated recurrent units have gradually been introduced into groundwater-level prediction. Among these methods, recurrent neural networks and their variants can capture dynamic dependencies in groundwater-level sequences and are well suited for hydrological time-series prediction with continuous temporal variations.^9^^,^^10^^,^^11^^,^^12^^,^^13^^,^^14^^,^^15^^,^^16^^,^^17^^,^^18^ The GRU model uses gating mechanisms to regulate the retention and updating of historical information. It maintains strong temporal learning capability while requiring relatively few parameters and offering high training efficiency.^19^^,^^20^^,^^21^ Therefore, GRU and its variants have considerable potential for groundwater-level prediction.

Although deep learning models have achieved promising performance in groundwater-level prediction, several limitations remain in existing studies. First, most models mainly rely on statistical relationships between input variables and groundwater levels, while constraints on groundwater dynamic processes and pumping-response mechanisms remain insufficient. When pumping intensity changes, recharge conditions fluctuate, or groundwater-level dynamics during the prediction period differ from those in the training period, these models may suffer from insufficient physical consistency and reduced generalizability. In addition, predictions from data-driven networks often lack physical interpretability, which limits their credibility in practical water resources management decisions.^22^

Physics-informed neural networks (PINNs) and physics-guided machine learning have provided new opportunities to improve the reliability of data-driven models. By incorporating physical constraints, such as governing equations, boundary conditions, or initial conditions, into neural network training, PINNs allow models to fit observations while satisfying prescribed physical laws as closely as possible. The PINN framework proposed by Raissi et al. has been widely used to solve forward problems and identify inverse problems in systems described by nonlinear partial differential equations.^23^ Subsequently, this concept was further extended by Karniadakis et al. into a broader physics-informed machine learning framework, in which observational data, mathematical models, and prior knowledge are integrated to improve model generalizability under data-limited and complex-system conditions.^24^ In recent years, these methods have gradually been applied to groundwater and hydrological processes. Zhang et al. proposed the GW-PINN method to solve groundwater flow equations with well source-sink terms.^25^ Secci et al. applied PINNs to transient groundwater flow equations in unconfined aquifers.^26^ Ali et al. systematically summarized the potential advantages of PINNs in groundwater flow modeling, including mesh-free computation, continuous spatiotemporal representation, and computational efficiency.^27^ Ji et al. combined PINNs with the KLE expansion for groundwater inverse problems.^28^ Yuan et al. further applied an improved PINN to pumping-well-induced groundwater flow simulations in homogeneous and heterogeneous aquifers.^29^ These studies indicate that embedding physical equations or simplified process constraints into neural network training has become an important direction for improving the reliability of hydrological and groundwater modeling.

However, existing physics-constrained groundwater studies are not fully aligned with the requirements of groundwater-level time-series prediction in agricultural pumping areas. On the one hand, many PINN-based studies have mainly focused on solving groundwater flow equations, developing surrogate models for numerical simulations, estimating parameters, or simulating flow fields under idealized pumping conditions. Their objectives are therefore closer to groundwater flow process simulation than to groundwater-level sequence prediction for actual monitoring wells. On the other hand, existing groundwater-level prediction studies have mostly focused on precipitation-driven water-level fluctuations. Jeong et al. embedded a linear reservoir model into a GRU network as a simplified physics-based constraint for precipitation-driven groundwater-level prediction, demonstrating the role of simplified physical constraints in improving model stability and prediction accuracy.^30^ However, in plain areas that have long been affected by agricultural irrigation pumping, groundwater-level variations are influenced not only by meteorological conditions, such as precipitation and evapotranspiration, but also by the sustained effects and cumulative impacts of pumping. Therefore, further research is needed on how agricultural pumping disturbances can be explicitly incorporated into groundwater-level prediction models.^31^^,^^32^^,^^33^

To address the insufficient incorporation of process-based prior knowledge and the inadequate characterization of differences in shallow and deep groundwater responses under agricultural pumping disturbances, a process-guided GRU framework for groundwater-level prediction that accounts for pumping disturbances and aquifer-specific response differences was developed in this study (Figure 1). Four shallow and four deep groundwater monitoring wells in a typical agricultural groundwater-extraction area of the North China Plain were selected (Figures 2 and 3; Table 1). To account for the distinct response characteristics of shallow and deep groundwater levels, differentiated process-guidance equations were formulated for the two aquifer groups to evaluate the contributions of process guidance and pumping-disturbance information to groundwater-level prediction performance in different aquifers. Given the limited number of monitoring wells included in this study, the present work should be regarded as a site-specific demonstration rather than a groundwater prediction framework that can be directly and broadly transferred to other regions. Nevertheless, this study may provide a process-guided modeling perspective and reference approach for groundwater-level prediction in agricultural groundwater-extraction areas.

**Figure 1:**
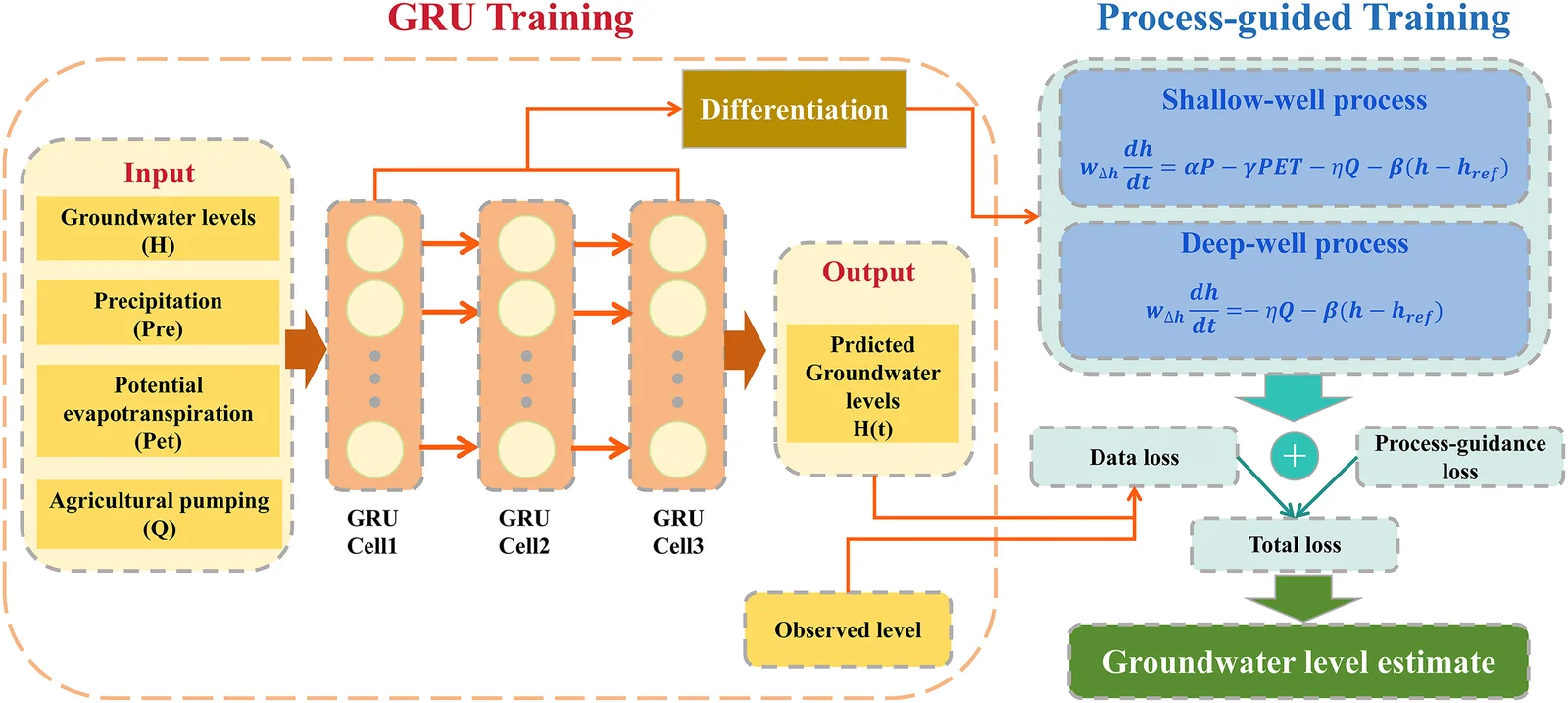
Schematic architecture of the process-guided GRU model for shallow and deep wells

**Figure 2:**
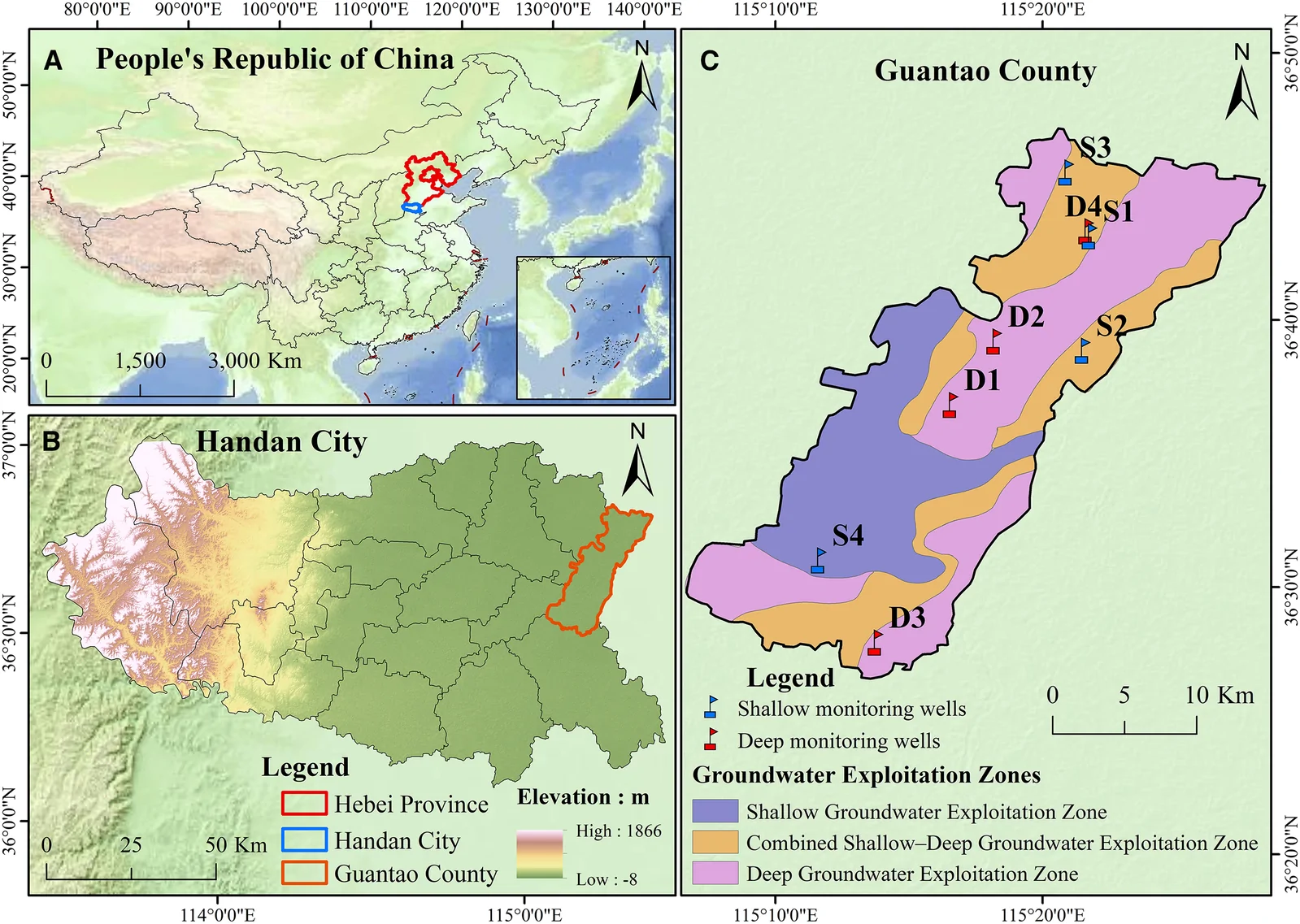
Geographic location of the study area and groundwater exploitation zones (A) Location of the study area in China. (B) Location of Guantao County within Handan City. (C) Distribution of shallow and deep monitoring wells and groundwater exploitation zones in Guantao County. S1, S2, S3, and S4 denote shallow monitoring wells, whereas D1, D2, D3, and D4 denote deep monitoring wells. The scale bars span 0–3,000 km in (A), 0–50 km in (B), and 0–10 km in (C), respectively.

**Figure 3:**
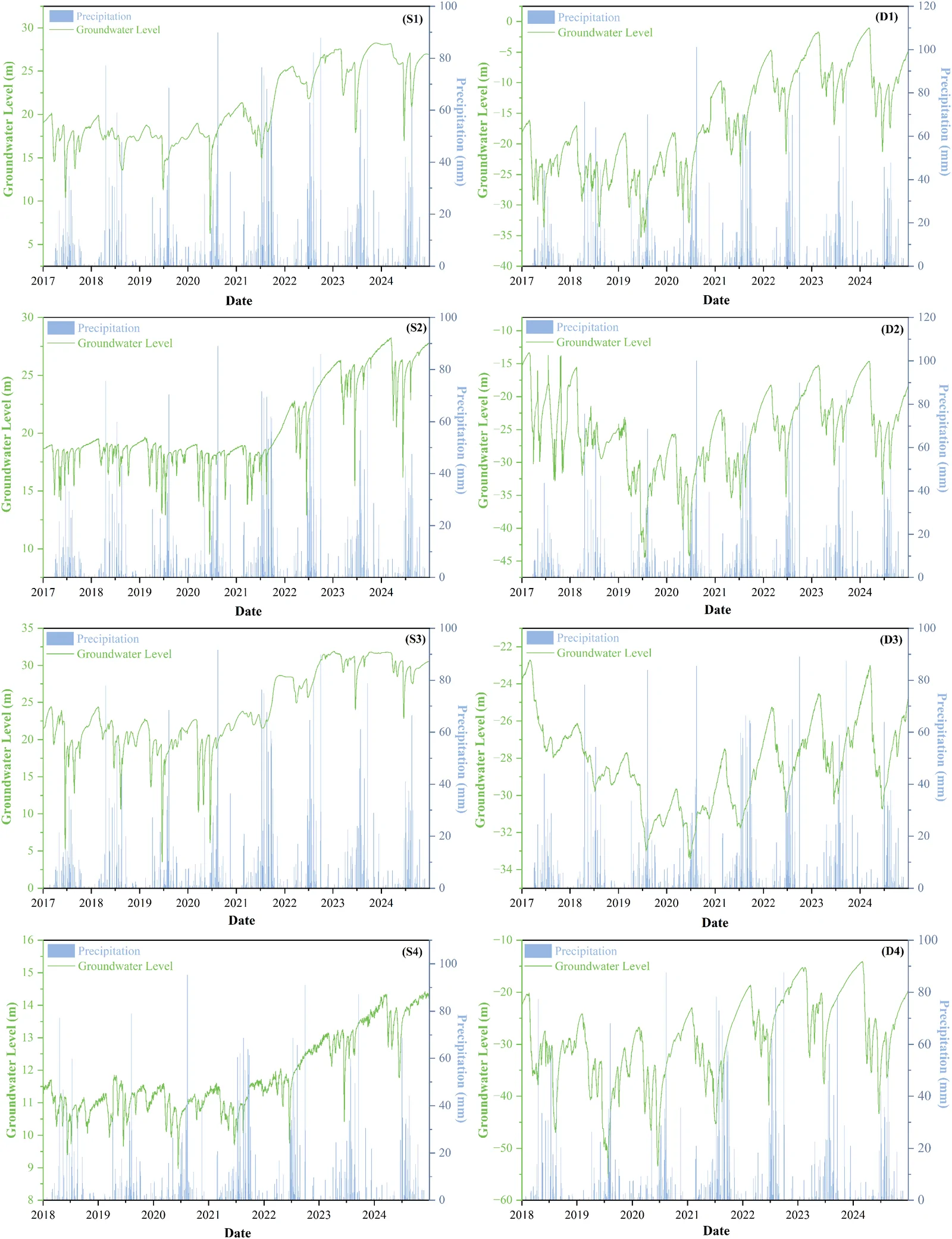
Groundwater-level and precipitation series for monitoring wells S1, S2, S3, S4, D1, D2, D3, and D4

**Table 1:** Data timestamps

Timeline	Monitoring well	Data volume
2017.01.01–2024.12.31	S1	2922
	S2	
	S3	
	D1	
	D2	
	D3	
2018.01.01–2024.12.31	S4	2557
	D4	

## Results

### Daily-scale groundwater-level prediction results

Figure 4 presents the daily-scale groundwater-level predictions produced by the GRU and PG-GRU models for four shallow and four deep monitoring wells (Table 2). Overall, both models reproduced the long-term groundwater-level trends and dynamic variations during the test period. The training loss decreased rapidly during the early stages of training and gradually stabilized, indicating that the training process had essentially converged. Compared with the GRU model, the PG-GRU predictions were generally closer to the observations for most monitoring wells during the test period. In particular, during periods of rapid groundwater-level change, the process-guided model showed an improved ability to track groundwater-level dynamics.

**Figure 4:**
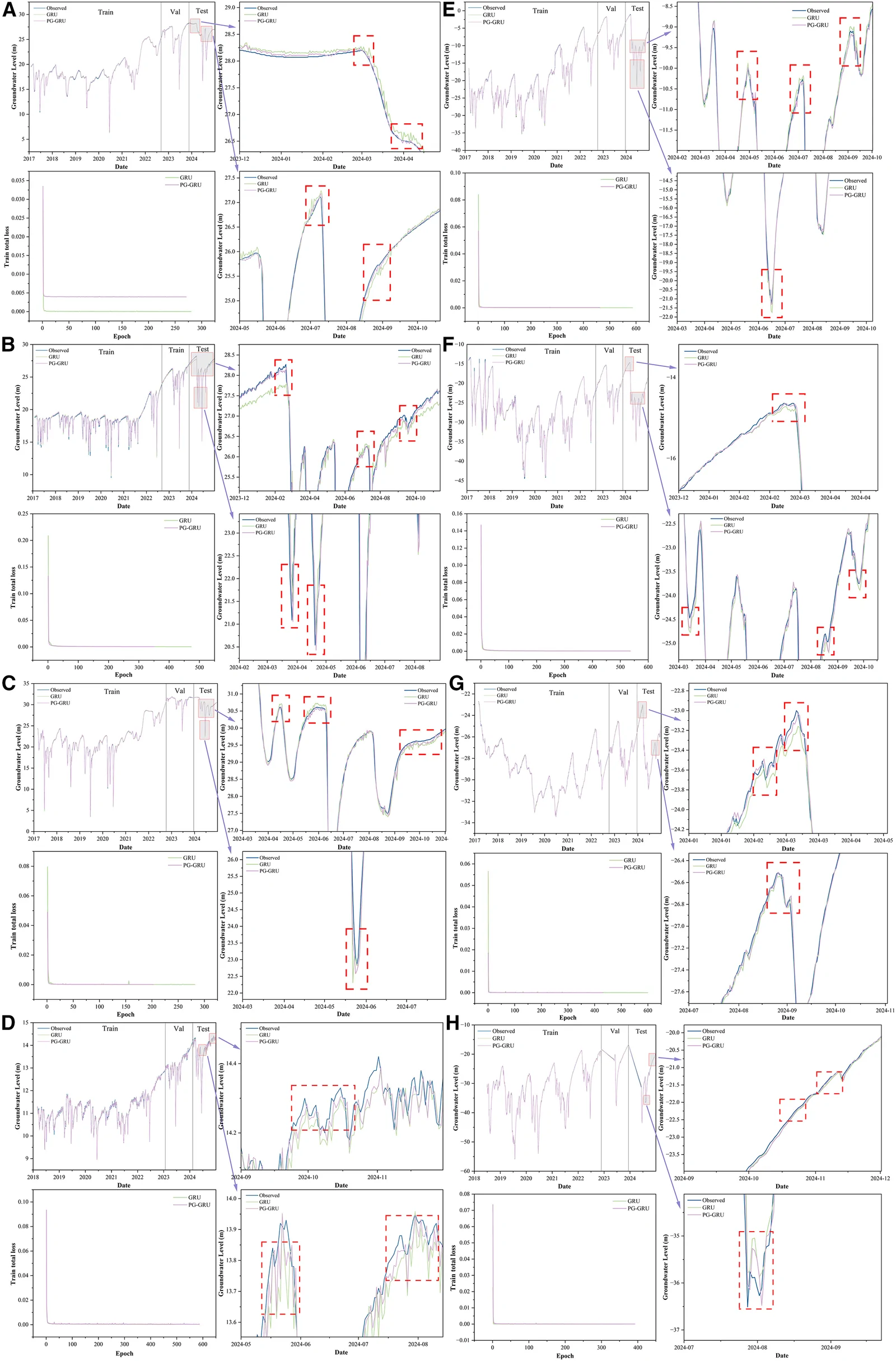
Daily-scale prediction results for eight monitoring wells under the GRU and PG-GRU models (A–H) S1, S2, S3, S4, D1, D2, D3, and D4, respectively. Each image includes the full-series prediction, the training-loss curve, and an enlarged view of the test-period predictions.

**Table 2:** Training settings and hyperparameter search space

Parameter	Daily scale	Dekadal (10-day) scale
Optimizer	AdamW	
Data partition	70%/15%/15%	
Optuna trials	80	
Maximum epochs (search)	800	500
Maximum epochs (final training)	1500	800
Early-stopping patience	120	80
Early-stopping criterion	validation loss	
Learning rate	1 × 10^−4^–5×10^−3^	1 × 10^−4^–3×10^−3^
Lookback	30, 60, 90, 120, 180 days	3, 6, 9, 12 dekads
Hidden size	32, 64, 96, 128	16, 32, 64
GRU layers	1–3	1–2
Dropout	0–0.30	
Batch size	32, 64, 128	16, 32
Weight decay	1 × 10^−7^–1×10^−3^	1 × 10^−6^–1×10^−3^
*w*_Δ*h*_	0.05–2.00	
α	0–2.00	
γ	0–2.00	
η	0–2.00	
β	0–2.00	
Process-loss weight	1 × 10^−4^–1.0	1 × 10^−6^–0.01
Random seeds	42, 52, 62, 72, 82	

The enlarged regions highlighted by the red dashed boxes show that the PG-GRU predictions were closer to the observed groundwater-level curves during several periods of rapid decline, recovery, or short-term fluctuation. These highlighted intervals mainly correspond to local abrupt changes or short-term variations in groundwater levels during the test period. The conventional GRU tended to underestimate or overestimate groundwater levels during some of these periods, whereas the PG-GRU tracked the local variations more consistently. These results indicate that, at the daily scale, introducing process-residual guidance allowed the model to preserve its ability to reproduce long-term trends while also improving, to some extent, its representation of local groundwater-level dynamics.

Based on test-set evaluation metrics, the PG-GRU model improved mean RMSE and NSE for both shallow and deep wells at the daily scale. For the shallow wells, the GRU model yielded a mean RMSE of 0.1967, a KGE of 0.9612, and an NSE of 0.9771, whereas the PG-GRU reduced the mean RMSE to 0.1825, with corresponding KGE and NSE values of 0.9668 and 0.9802, respectively. Compared with the GRU model, the mean RMSE of the PG-GRU for the shallow wells was reduced by approximately 7.2%, indicating that the incorporation of process-residual guidance improved the model’s ability to reproduce daily groundwater-level variations in the shallow aquifer. However, KGE and Bias did not improve consistently at every monitoring well, indicating that the benefits of process guidance showed some well-specific dependence.

At the individual-well level, differences in prediction difficulty were observed among the four shallow wells. At the daily scale, the RMSE for well S2 was markedly higher than those for S1, S3, and S4. For example, the GRU model yielded an RMSE of 0.3914 for S2, compared with 0.1448, 0.1559, and 0.0947 for S1, S3, and S4, respectively. Similarly, the PG-GRU model produced an RMSE of 0.3578 for S2, which remained higher than those for the other three shallow wells. This suggests that groundwater-level dynamics at S2 may exhibit stronger local variability or more complex relationships with the driving factors, resulting in greater prediction difficulty. Nevertheless, the RMSE obtained by the PG-GRU was lower than that of the GRU for all four shallow wells, indicating a relatively consistent improvement in reducing prediction errors for the shallow wells through process guidance.

For the deep wells, overall prediction accuracy was high at the daily scale. The GRU model yielded a mean RMSE of 0.1632, a KGE of 0.9949, and an NSE of 0.9987, whereas the PG-GRU further reduced the mean RMSE to 0.1533, with corresponding KGE and NSE values of 0.9951 and 0.9988, respectively. Compared with the GRU model, the mean RMSE of the PG-GRU for the deep wells was reduced by approximately 6.1%. However, compared with the shallow wells, the differences in evaluation metrics among the models were smaller for the deep wells. This suggests that deep groundwater levels may exhibit stronger temporal persistence and inertia at the daily scale, allowing the conventional GRU to capture most of the short-term variations.

Overall, the daily-scale results showed that the PG-GRU generally achieved lower RMSE and higher NSE values for both shallow and deep wells. Incorporating process-residual guidance helped reduce prediction errors and, to some extent, improved the model’s representation of groundwater-level dynamics. However, the magnitude of improvement was not entirely consistent across different evaluation metrics or monitoring wells. These results indicate that embedding prior knowledge of groundwater-level dynamics into GRU training can incrementally improve daily groundwater-level prediction under agricultural pumping disturbances.

### Dekadal-scale groundwater-level prediction results

Figure 5 presents the groundwater-level predictions obtained using the GRU and PG-GRU models at the dekadal scale. Overall, both models were able to reproduce the main groundwater-level trends, and the predicted and observed series generally showed consistent patterns during the training, validation, and test periods. Compared with the daily scale, short-term fluctuations in the groundwater-level series were partially smoothed by temporal aggregation at the dekadal scale, and groundwater-level variations were more prominently expressed as periods of rise and decline. Accordingly, model performance at this scale was mainly reflected in the ability to reproduce these temporal trends and the positions of local peaks and troughs.

**Figure 5:**
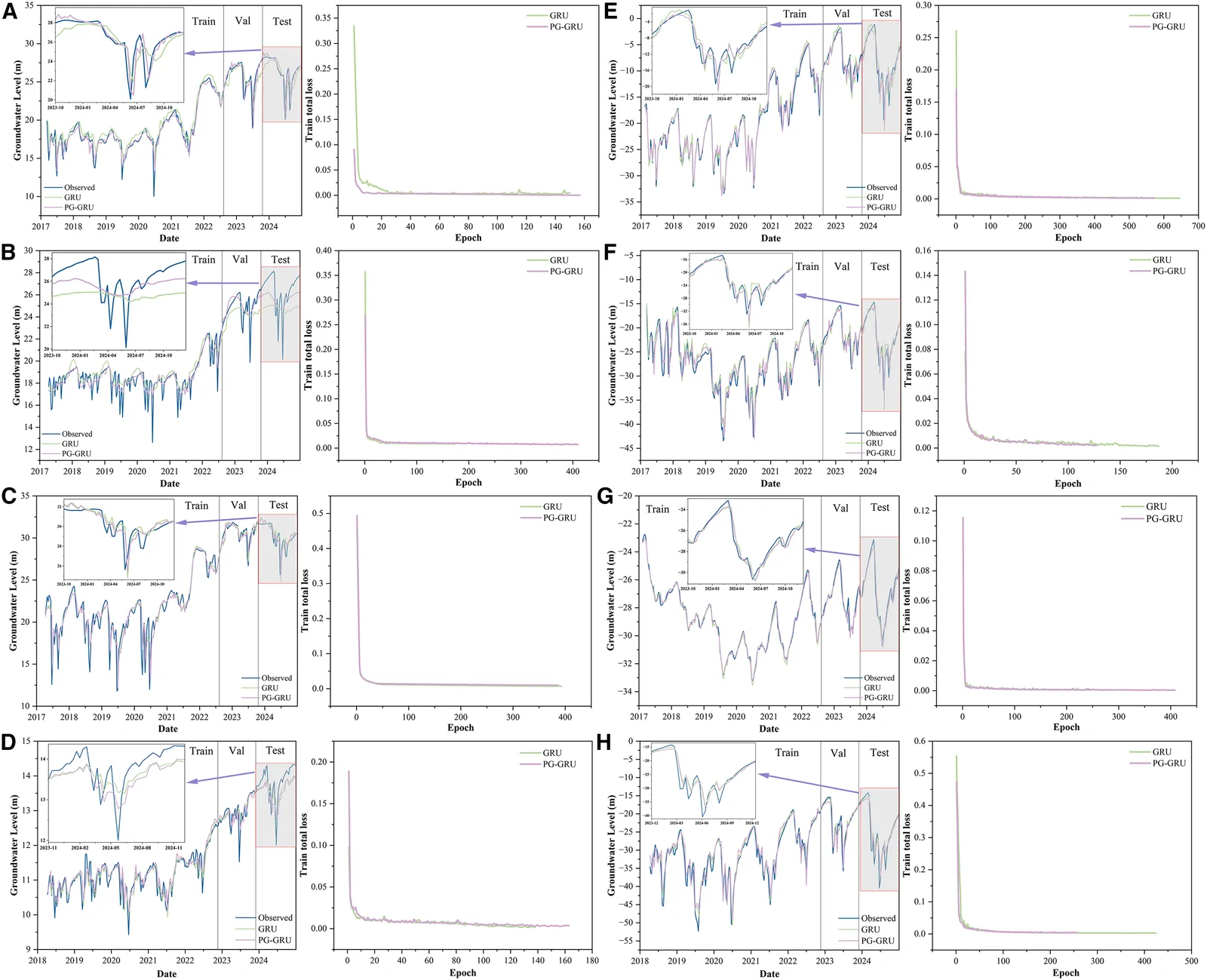
Dekadal-scale prediction results for eight monitoring wells under the GRU and PG-GRU models (A–H) S1, S2, S3, S4, D1, D2, D3, and D4, respectively. Each image includes the full-series prediction, an enlarged view of the test-period predictions, and the training-loss curve.

The enlarged views of the test period show that the PG-GRU predictions were closer to the observations during several intervals for S1, S3, S4, and D1–D4, particularly during periods of groundwater-level rise or decline and around local peaks and troughs, where the prediction deviations were relatively smaller. For S2, neither model was able to fully capture the groundwater-level variations during the test period. Although the PG-GRU showed slight improvements over the GRU during some intervals, the overall prediction error remained relatively large. This indicates that process guidance can still provide some improvement at the dekadal scale, although its effectiveness is influenced by the complexity of well-specific groundwater dynamics.

For the shallow wells, the GRU model yielded a mean RMSE of 1.0307, a KGE of 0.5781, and an NSE of 0.3894, whereas the PG-GRU reduced the mean RMSE to 0.9890 and increased the NSE and KGE to 0.4419 and 0.6060, respectively. Compared with the GRU model, the mean RMSE of the PG-GRU for shallow wells decreased by about 4.0%, indicating that process guidance still provided limited improvement at the dekadal scale, although the improvement was smaller than at the daily scale. Notably, for S2, the PG-GRU yielded an NSE of −0.0020 and a KGE of 0.1531 at the dekadal scale. Although the NSE was improved relative to that of the GRU model (−0.0806), the overall predictive performance remained weak.

For the deep wells, the GRU model yielded a mean RMSE of 1.7120, a KGE of 0.9014, and an NSE of 0.8622, whereas the PG-GRU reduced the mean RMSE to 1.6756 and increased the NSE to 0.8687, with the KGE increasing slightly to 0.9034. Compared with the GRU model, the mean RMSE was reduced by approximately 2.1%. Although the magnitude of improvement was small, the PG-GRU still showed modest improvements in the group-mean RMSE and NSE, indicating a limited positive effect of process guidance on dekadal-scale groundwater-level prediction for the deep wells.

Overall, the PG-GRU showed limited improvements in the group-mean performance metrics at the dekadal scale. However, the improvement was smaller than at the daily scale and was not fully consistent across monitoring wells. As the temporal resolution changed from daily to dekadal, short-term driving information and local response characteristics in groundwater-level variations may have been weakened, thereby reducing the extent to which process guidance improved prediction accuracy.

### Model performance differences across timescales

To further evaluate predictive performance across different model types, Table 3 also reports the test-set results for MLR, SVR, GRU, and PG-GRU. At the daily scale, both MLR and SVR were able to reproduce the overall groundwater-level variations reasonably well; however, their mean RMSE values for the shallow wells were 0.2579 and 0.3916, respectively, both of which were higher than those of the GRU (0.1967) and PG-GRU (0.1825). A similar pattern was observed for the deep wells, for which the PG-GRU achieved the lowest mean RMSE. At the dekadal scale, prediction errors for MLR and SVR generally increased further. For the shallow wells, the mean RMSE values were 1.6582 and 1.1572 for MLR and SVR, respectively, compared with 1.0307 for the GRU and 0.9890 for the PG-GRU. For the deep wells, the PG-GRU likewise achieved the lowest mean RMSE.

**Table 3:** Test-set groundwater-level prediction results obtained using the MLR, SVR, GRU, and PG-GRU models: (a) daily-resolution data and (b) dekadal-resolution data

	S1	S2	S3	S4	Mean	Std	D1	D2	D3	D4	Mean	Std
**(a) Daily**												

**MLR model**												
RMSE	0.2362	0.4497	0.1906	0.1551	0.2579	0.1321	0.2885	0.2852	0.0905	0.2807	0.2362	0.0972
KGE	0.9932	0.9653	0.9894	0.9573	0.9763	0.0177	0.9937	0.9975	0.9972	0.9966	0.9962	0.0018
NSE	0.9865	0.9357	0.9838	0.9420	0.9620	0.0269	0.9961	0.9962	0.9978	0.9984	0.9972	0.0011
Bias	0.0088	−0.1977	−0.0356	−0.1086	−0.0833	0.0903	−0.0481	−0.0311	−0.0088	0.0137	−0.0186	0.0269
SVR model												
RMSE	0.2584	0.8737	0.2204	0.2137	0.3916	0.3220	0.3009	0.2879	0.0902	0.2844	0.2408	0.1007
KGE	0.9871	0.8157	0.9734	0.9328	0.9272	0.0779	0.9839	0.9963	0.9967	0.9969	0.9935	0.0064
NSE	0.9839	0.7574	0.9783	0.8233	0.8857	0.1134	0.9958	0.9962	0.9978	0.9984	0.9970	0.0013
Bias	−0.0554	−0.6022	−0.0742	−0.1917	−0.2309	0.2548	0.0073	0.0538	−0.0005	−0.0035	0.0143	0.0267
**GRU model**												
RMSE	0.1448	0.3914	0.1559	0.0947	0.1967	0.1325	0.1580	0.1798	0.0660	0.2490	0.1632	0.0755
KGE	0.9859	0.9338	0.9639	0.9614	0.9612	0.0213	0.9969	0.9964	0.9927	0.9934	0.9949	0.0021
NSE	0.9950	0.9544	0.9886	0.9703	0.9771	0.0184	0.9989	0.9986	0.9990	0.9982	0.9987	0.0004
Bias	0.0290	−0.0946	−0.0039	−0.0074	−0.0192	0.0528	0.0164	−0.0056	−0.0094	0.0020	0.0008	0.0114
**PG-GRU model**												
RMSE	0.1360	0.3578	0.1458	0.0905	0.1825	0.1193	0.1414	0.1687	0.0614	0.2419	0.1533	0.0746
KGE	0.9787	0.9743	0.9679	0.9465	0.9668	0.0143	0.9967	0.9955	0.9957	0.9926	0.9951	0.0018
NSE	0.9956	0.9619	0.9905	0.9729	0.9802	0.0156	0.9991	0.9988	0.9990	0.9983	0.9988	0.0004
Bias	−0.0207	−0.0658	−0.0028	−0.0160	−0.0263	0.0274	−0.0053	0.0143	0.0052	−0.0029	0.0028	0.0089

**(b) Dekadal (10-day)**												

**MLR model**												
RMSE	1.2795	3.0842	1.5917	0.6775	1.6582	1.0235	2.2671	2.0388	0.5602	2.6456	1.8779	0.9134
KGE	0.8130	0.5613	0.7667	0.7431	0.7210	0.1104	0.8412	0.8954	0.9545	0.9046	0.8989	0.0464
NSE	0.5505	−2.6537	−0.2532	−0.9862	−0.8357	1.3649	0.7539	0.8009	0.9130	0.8596	0.8318	0.0693
Bias	−0.5249	−2.8401	−1.2556	−0.6015	−1.3055	1.0743	−1.0192	−0.6592	−0.0764	−0.7040	−0.6147	0.3930
**SVR model**												
RMSE	1.3259	1.6726	1.2696	0.3607	1.1572	0.5601	2.0477	1.9896	0.5379	2.9215	1.8742	0.9876
KGE	0.7773	0.6049	0.6554	0.7544	0.6980	0.0815	0.8744	0.9059	0.9450	0.9033	0.9072	0.0290
NSE	0.5173	0.1114	0.2026	0.4369	0.3171	0.1914	0.7992	0.8104	0.9198	0.8288	0.8396	0.0549
Bias	0.3024	−0.2843	0.3499	−0.1262	0.0605	0.3141	−0.4302	0.1675	−0.0412	−0.5275	−0.2078	0.3267
**GRU model**												
RMSE	1.1691	1.6511	0.9512	0.3512	1.0307	0.5392	2.0436	1.8643	0.4693	2.4709	1.7120	0.8667
KGE	0.7776	0.1676	0.7682	0.5988	0.5781	0.2857	0.8864	0.9004	0.9639	0.8550	0.9014	0.0458
NSE	0.6224	−0.0806	0.5520	0.4638	0.3894	0.3200	0.7996	0.8329	0.9389	0.8775	0.8622	0.0602
Bias	0.1532	−0.8213	0.2926	−0.1470	−0.1306	0.4957	0.0871	0.0406	−0.0668	0.5275	0.1471	0.2616
**PG-GRU model**												
RMSE	1.0932	1.6093	0.9225	0.3313	0.9890	0.5268	1.9701	1.8208	0.4654	2.4460	1.6756	0.8497
KGE	0.7864	0.1531	0.7862	0.6982	0.6060	0.3047	0.8955	0.8942	0.9639	0.8602	0.9034	0.0435
NSE	0.6714	−0.0020	0.5785	0.5198	0.4419	0.3025	0.8140	0.8409	0.9398	0.8799	0.8687	0.0546
Bias	0.1129	−0.7710	0.2330	−0.1134	−0.1346	0.4479	−0.1975	0.0322	−0.0602	0.3703	0.0362	0.2419

Taken together, the daily- and dekadal-scale results indicate that model performance was sensitive to temporal resolution. At the daily scale, the groundwater-level series retained more complete information on continuous temporal variations and short-term fluctuations. Under these conditions, the PG-GRU achieved lower mean RMSE and higher NSE values than the GRU for both shallow and deep wells. By contrast, at the dekadal scale, temporal aggregation smoothed part of the short-term fluctuations and local response signals, thereby reducing the performance differences among the models. At this coarser temporal scale, the benefits of the PG-GRU were reflected primarily in modest reductions in overall prediction error and limited improvements in the process-oriented performance metrics.

The sensitivity to temporal aggregation also differed between shallow and deep wells. Prediction accuracy for the shallow wells was relatively high at the daily scale, whereas KGE and NSE declined more markedly at the dekadal scale. This suggests that shallow groundwater-level variations contain a greater proportion of short-term fluctuations and local disturbance signals, which may be weakened by temporal aggregation. In contrast, the deep wells exhibited high overall predictive accuracy at the daily scale and retained relatively good process-reproduction performance at the dekadal scale, indicating that deep groundwater-level variations still showed relatively strong temporal continuity at the coarser resolution.

Therefore, model evaluation at a single temporal scale is insufficient to fully characterize the applicability of the process-guided model. The daily-scale results more clearly reflect the corrective effect of process guidance on short-term groundwater-level dynamics, whereas the dekadal-scale results better represent the model’s ability to reproduce longer-term temporal trends. Collectively, the results at both scales show that the performance gains of the PG-GRU were not consistent across all temporal scales, monitoring wells, or evaluation metrics, but were jointly influenced by temporal resolution, aquifer type, and well-specific groundwater dynamics.

Based on these cross-scale differences, two aspects require further discussion. First, the contribution of groundwater-pumping information to predictive performance for the shallow and deep wells should be further evaluated. Second, for the deep wells, the use of pumping data at a single daily scale may be insufficient to represent cumulative groundwater responses, and model performance under different cumulative pumping windows therefore needs to be further compared.

## Discussion

### Effect of pumping information on model prediction performance

To identify the contribution of pumping-disturbance information to model performance, the full PG-GRU model was further compared with a control model, PG-GRU-noQ, in which the pumping term was removed from the process residual. Both models used the same GRU prediction backbone and the same basic form of the process residual. The only difference was that the pumping term was included in the process residual of the PG-GRU but omitted from that of the PG-GRU-noQ. Therefore, the performance difference between the two models was used to assess the additional contribution of pumping information when explicitly incorporated as a process-guidance term. It should be noted that the PG-GRU-noQ did not completely remove process guidance; rather, the basic process-residual structure corresponding to each well type was retained, while the pumping-disturbance term was excluded.

As shown in Figure 6 and Table 4, model performance for the shallow wells was generally improved after the pumping term was incorporated. At the daily scale, the PG-GRU achieved a mean RMSE of 0.1825, lower than those of the GRU (0.1967) and PG-GRU-noQ (0.2152). Its mean KGE and NSE were 0.9668 and 0.9802, respectively, both higher than those of the PG-GRU-noQ (0.9533 and 0.9687). After the Q term was removed from the process residual, the RMSE increased for the shallow wells, while the absolute mean Bias increased from 0.0263 for the PG-GRU to 0.0459 for the PG-GRU-noQ. These results indicate that explicitly incorporating pumping information as a process-guidance term helped the model better represent shallow groundwater-level dynamics under agricultural pumping disturbances. At the dekadal scale, the mean RMSE of the PG-GRU for the shallow wells was 0.9890, lower than those of the GRU (1.0307) and PG-GRU-noQ (1.0642). The NSE also increased from 0.3894 for the GRU to 0.4419 for the PG-GRU, whereas the PG-GRU-noQ yielded an NSE of 0.3780. Although the mean KGE of the PG-GRU-noQ (0.6227) was slightly higher than that of the PG-GRU (0.6060), the PG-GRU performed better in terms of RMSE and NSE.

**Figure 6:**
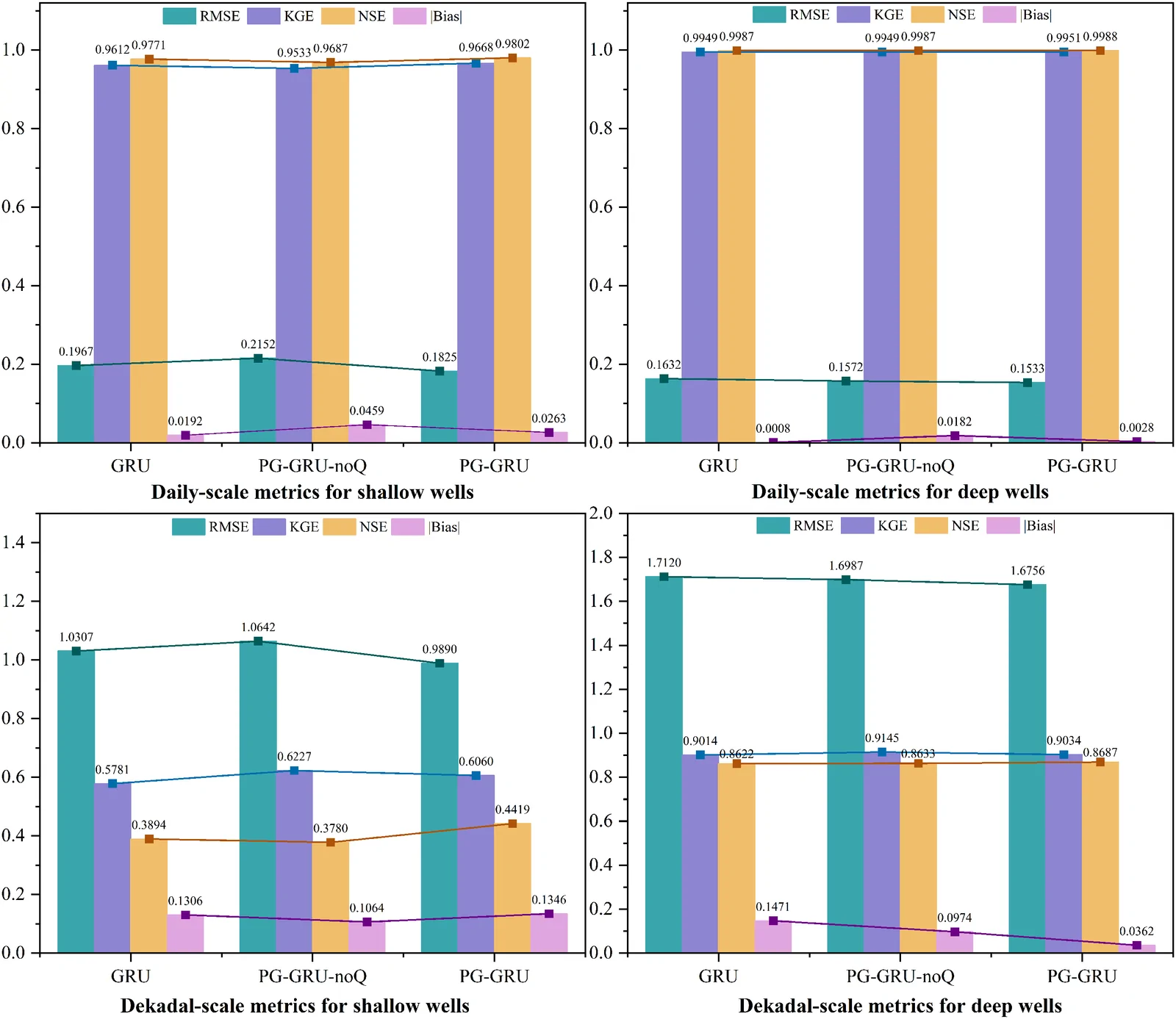
Comparison of RMSE, KGE, NSE, and Bias for the GRU, PG-GRU-noQ, and PG-GRU models for shallow and deep wells at the daily and dekadal scales

**Table 4:** Results of the PG-GRU-noQ model

	S1	S2	S3	S4	Mean	Std	D1	D2	D3	D4	Mean	Std
**(a) Daily**												

RMSE	0.1422	0.4218	0.1781	0.1189	0.2152	0.1398	0.1405	0.1733	0.0625	0.2524	0.1572	0.0787
KGE	0.9844	0.9234	0.9454	0.9602	0.9533	0.0257	0.9967	0.9950	0.9962	0.9917	0.9949	0.0023
NSE	0.9952	0.9499	0.9838	0.9457	0.9687	0.0246	0.9991	0.9987	0.9990	0.9982	0.9987	0.0004
Bias	−0.0181	−0.1390	0.0129	−0.0396	−0.0459	0.0657	−0.0067	0.0141	−0.0015	0.0667	0.0182	0.0336

**(b) Dekadal (10-day)**												

RMSE	1.3409	1.6374	0.9513	0.3272	1.0642	0.5660	2.0782	1.8252	0.4758	2.4156	1.6987	0.8504
KGE	0.7834	0.2554	0.7654	0.6864	0.6227	0.2484	0.8902	0.9070	0.9626	0.8982	0.9145	0.0328
NSE	0.4984	−0.0716	0.5517	0.5334	0.3780	0.3005	0.7929	0.8403	0.9371	0.8829	0.8633	0.0614
Bias	0.3795	−0.9637	0.2865	−0.1278	−0.1064	0.6126	0.0432	0.0840	−0.0157	0.2780	0.0974	0.1272

It should also be noted that the overall predictive performance of the shallow wells at the dekadal scale was substantially weaker than that at the daily scale. In particular, the PG-GRU yielded an NSE of −0.0020 for S2, indicating limited ability to reproduce the aggregated groundwater-level dynamics at this well. Temporal aggregation may smooth high-frequency fluctuations while simultaneously weakening short-term response information among precipitation, pumping, and groundwater levels and reducing the effective sample size. Therefore, the improvement provided by the pumping-related process-guidance term at the dekadal scale should be interpreted as a limited and strongly well-dependent gain rather than evidence of consistently high predictive accuracy across all shallow wells. Taken together, the daily- and dekadal-scale results indicate that the pumping term made a relatively consistent positive contribution to shallow-well prediction, particularly in reducing prediction errors and improving the reproduction of groundwater-level dynamics.

For deep wells, including the pumping term also improved performance, although its contribution differed from that observed for shallow wells. At the daily scale, the PG-GRU achieved a mean RMSE of 0.1533, lower than those of the PG-GRU-noQ (0.1572) and GRU (0.1632). The NSE values of all three models were approximately 0.999, and their KGE values were all close to 0.995. At the dekadal scale, the mean RMSE of the PG-GRU was 1.6756, lower than those of the GRU (1.7120) and PG-GRU-noQ (1.6987). Its NSE was 0.8687, also slightly higher than those of the GRU (0.8622) and PG-GRU-noQ (0.8633). Although the PG-GRU-noQ yielded a higher KGE (0.9145) than the PG-GRU (0.9034) at the dekadal scale, the PG-GRU still showed better overall error control and process reproduction in terms of RMSE, NSE, and Bias after the cumulative pumping-disturbance term was incorporated. These results suggest that, for the deep wells, the pumping-related process-guidance term contributed primarily to correcting prediction errors under cumulative pumping disturbances, rather than producing the more evident simultaneous improvement across multiple metrics observed for the shallow wells.

Overall, pumping-disturbance information contributed more directly to shallow wells, whereas for deep wells it was mainly reflected in prediction-error correction under cumulative pumping disturbances. This difference may be related to the more direct response of shallow groundwater to agricultural pumping and near-surface hydrological processes, whereas deep groundwater levels may respond to pumping disturbances with greater lag and cumulative effects. Therefore, in groundwater-level prediction for agricultural pumping areas, the role of pumping information as a process-guidance term should be evaluated according to aquifer type and prediction timescale rather than treated in a uniform manner.

### Sensitivity analysis of cumulative pumping time windows for deep wells

Considering that deep groundwater levels typically exhibit delayed and cumulative responses to pumping disturbances, the effects of 30-, 60-, and 90-day moving-average pumping series on deep-well prediction were further compared to evaluate how effectively pumping disturbances were represented under different accumulation windows.

The daily-scale results (Table 5) showed that the effects of different pumping windows on predictive performance varied among the deep wells. In terms of RMSE, D1 and D3 achieved their lowest errors under the 90-day window, with RMSE values of 0.1384 and 0.0605, respectively. D2 performed best under the 60-day window, with an RMSE of 0.1687, whereas D4 achieved its lowest RMSE of 0.2071 under the 30-day window. Across the four deep wells, the lowest mean RMSE was obtained with the 30-day window (0.1454), followed by the 60-day (0.1533) and 90-day (0.1616) windows. These results indicate clear well-specific differences in the response of deep groundwater to cumulative pumping disturbances, and no single accumulation window produced the best predictive performance for all wells.

**Table 5:** Prediction metrics for deep wells under different temporal windows at the daily scale

	D1	D2	D3	D4	Mean	Std
**GRU model**						

RMSE	0.1580	0.1798	0.0660	0.2490	0.1632	0.0755
KGE	0.9969	0.9964	0.9927	0.9934	0.9949	0.0021
NSE	0.9989	0.9986	0.9990	0.9982	0.9987	0.0004
Bias	0.0164	−0.0056	−0.0094	0.0020	0.0008	0.0114

**PG-GRU model**						

**(a) 60 days**						
RMSE	0.1414	0.1687	0.0614	0.2419	0.1533	0.0746
KGE	0.9967	0.9955	0.9957	0.9926	0.9951	0.0018
NSE	0.9991	0.9988	0.9990	0.9983	0.9988	0.0004
Bias	−0.0053	0.0143	0.0052	−0.0029	0.0028	0.0089
**(b) 30 days**						
RMSE	0.1393	0.1746	0.0606	0.2071	0.1454	0.0629
KGE	0.9958	0.9961	0.9961	0.9910	0.9948	0.0025
NSE	0.9991	0.9987	0.9991	0.9988	0.9989	0.0002
Bias	0.0082	0.0327	0.0015	0.0146	0.0143	0.0134
**(c) 90 days**						
RMSE	0.1384	0.1736	0.0605	0.2740	0.1616	0.0886
KGE	0.9975	0.9941	0.9989	0.9838	0.9936	0.0068
NSE	0.9992	0.9987	0.9991	0.9978	0.9987	0.0006
Bias	−0.0076	0.0155	−0.0046	0.0308	0.0085	0.0181

At the dekadal scale (Table 6), the influence of the pumping window on model performance was more pronounced. D1 and D2 achieved their lowest RMSE values under the 60-day window, at 1.9701 and 1.8208, respectively. D3 achieved its lowest RMSE of 0.4482 under the 30-day window, whereas D4 performed best under the 90-day window, with an RMSE of 2.4367. Across the four deep wells, the 60-day window produced the lowest mean RMSE (1.6756), followed by the 30-day window (1.6840), while the 90-day window yielded the highest value (1.7220). Compared with the mean RMSE of the GRU baseline model (1.7120), both the 30- and 60-day windows reduced the prediction error, whereas the 90-day window produced a slightly higher RMSE than the GRU. This suggests that an excessively long smoothing window may weaken the temporal variations in the pumping-disturbance series, thereby reducing its correspondence with groundwater-level variations at the dekadal scale.

**Table 6:** Prediction metrics for deep wells under different temporal windows at the dekadal scale

	D1	D2	D3	D4	Mean	Std
**GRU model**						

RMSE	2.0436	1.8643	0.4693	2.4709	1.7120	0.8667
KGE	0.8864	0.9004	0.9639	0.8550	0.9014	0.0458
NSE	0.7996	0.8329	0.9389	0.8775	0.8622	0.0602
Bias	0.0871	0.0406	−0.0668	0.5275	0.1471	0.2616

**PG-GRU model**						

**(a) 60 days**						
RMSE	1.9701	1.8208	0.4654	2.4460	1.6756	0.8497
KGE	0.8955	0.8942	0.9639	0.8602	0.9034	0.0435
NSE	0.8140	0.8409	0.9398	0.8799	0.8687	0.0546
Bias	−0.1975	0.0322	−0.0602	0.3703	0.0362	0.2419
**(b) 30 days**						
RMSE	1.9716	1.8573	0.4482	2.4590	1.6840	0.8642
KGE	0.8994	0.8838	0.9712	0.8649	0.9048	0.0464
NSE	0.8137	0.8347	0.9443	0.8786	0.8678	0.0577
Bias	−0.0084	0.0791	−0.0138	0.4166	0.1184	0.2033
**(c) 90 days**						
RMSE	2.1143	1.8668	0.4704	2.4367	1.7220	0.8664
KGE	0.8888	0.8989	0.9587	0.8710	0.9043	0.0380
NSE	0.7853	0.8330	0.9386	0.8808	0.8594	0.0656
Bias	−0.0418	0.1755	0.0103	0.3451	0.1223	0.1751

These results indicate that the effective representation of pumping disturbances in deep wells is strongly dependent on the selected temporal window. Shorter windows preserve more of the temporal variability in pumping disturbances but may be insufficient to represent the cumulative effects of sustained pumping. Longer windows can enhance the representation of cumulative effects but may also oversmooth the pumping process and weaken part of the informative disturbance signal. The 30-day window produced the lowest four-well mean RMSE at the daily scale, whereas the 60-day window yielded the lowest mean RMSE at the dekadal scale, further demonstrating that the optimal window is dependent on both the monitoring well and the temporal scale.

Overall, the influence of pumping information on model performance depends not only on whether a pumping term is incorporated into the process residual, but also on whether the temporal scale of the pumping disturbance is represented appropriately. When the moving-average window is better matched to the groundwater-level response characteristics of a given well, the prediction error of the PG-GRU can be further reduced. Future studies could incorporate a larger number of deep monitoring wells, the spatial distribution of pumping wells, and aquifer-structure information to further identify well-specific response timescales to pumping disturbances and to develop more adaptive representations of cumulative pumping effects.

### Methodological novelty and implications

The process-guided GRU (PG-GRU) model proposed in this study was not developed by simply applying a GRU to groundwater-level prediction. Instead, a lumped process residual representing groundwater-level dynamics was incorporated into a data-driven time-series prediction framework. Unlike the conventional GRU, which relies solely on historical groundwater levels and external driving variables to establish statistical mappings, the proposed model incorporates groundwater-level continuity, meteorological recharge and depletion, pumping responses, and groundwater-level recovery into the training process, thereby allowing model fitting to be guided by simplified hydrological-process information. This design enables hydrological process knowledge to be introduced into an otherwise purely data-driven prediction framework and enhances the process-based interpretability of model predictions.

Compared with previous physics-constrained studies that have primarily focused on precipitation-driven groundwater-level fluctuations or solving groundwater-flow equations, a key feature of this study is the explicit incorporation of agricultural pumping disturbances into groundwater-level prediction, along with the formulation of differentiated process residuals for shallow and deep wells. For the shallow wells, precipitation recharge, potential evapotranspiration loss, daily pumping, and groundwater-level recovery were jointly considered. For the deep wells, because their direct responses to daily meteorological processes are relatively weak, cumulative pumping disturbances and groundwater-level recovery were emphasized. This differentiated process-guidance scheme is consistent with the distinct response characteristics of shallow and deep groundwater in the study area and enables groundwater-level dynamics in different aquifer types to be represented more specifically.

The results showed that the PG-GRU generally achieved lower RMSE and higher NSE values than the GRU at both the daily and dekadal scales, indicating that the process-guided residual can improve groundwater-level prediction to some extent. Based on the unified main-model results for the eight monitoring wells, the RMSE of the PG-GRU relative to the GRU was reduced by approximately 7.2% and 6.1% for the shallow and deep wells, respectively, at the daily scale, and by approximately 4.0% and 2.1%, respectively, at the dekadal scale. These results indicate that the performance gains provided by the PG-GRU were generally incremental. This was particularly evident for the deep wells, for which the conventional GRU already achieved high predictive accuracy, leaving relatively limited room for further improvement. The pumping term contributed more for the shallow wells, whereas for the deep wells, the effectiveness of pumping information depended more strongly on an appropriate representation of the cumulative response window. Sensitivity analysis of different pumping windows further showed that predictive performance for the deep wells depended not only on whether pumping information was included, but also on how the temporal scale of pumping disturbances was represented.

To further evaluate the practical significance of model improvement in terms of actual groundwater-level error, the group-mean RMSE values of the GRU and PG-GRU were calculated for different temporal scales and aquifer types, and both the absolute error reduction and relative improvement were quantified. For the deep wells, differences in pumping-response time windows were further considered in the calculation. As shown in Table 7, at the daily scale, the group-mean RMSE for the shallow wells decreased from 0.1967 m to 0.1825 m, corresponding to an absolute reduction of 1.42 cm. For the deep wells, the RMSE decreased from 0.1632 m to 0.1437 m, corresponding to an absolute reduction of 1.95 cm and a relative reduction of approximately 12.0%. At the dekadal scale, the group-mean RMSE for the shallow wells decreased from 1.0307 m to 0.9890 m, corresponding to an absolute reduction of 4.17 cm. For the deep wells, the RMSE decreased from 1.7120 m to 1.6690 m, corresponding to an absolute reduction of 4.31 cm and a relative reduction of approximately 2.5%. In magnitude, these improvements represent incremental gains in predictive accuracy, but they remain practically meaningful for groundwater management.

**Table 7:** Absolute and relative improvements in group-mean RMSE from GRU to PG-GRU

Temporal scale	Aquifer type	GRU RMSE (m)	PG-GRU RMSE (m)	Absolute reduction (cm)	Relative reduction (%)
Daily	Shallow	0.1967	0.1825	1.42	7.2
Daily	Deep	0.1632	0.1437	1.95	12.0
Dekadal (10-day)	Shallow	1.0307	0.9890	4.17	4.0
Dekadal (10-day)	Deep	1.7120	1.6690	4.31	2.5

Note: The group-mean RMSE for the deep-well PG-GRU was calculated using the lowest RMSE obtained for each well in the window-sensitivity analysis. At the daily scale, the selected windows for D1–D4 were 90, 60, 90, and 30 days, respectively; at the dekadal scale, the corresponding windows were 60, 60, 30, and 90 days.

Dynamic groundwater management depends on continuously identifying groundwater-level decline, recovery, turning points, and anomalous fluctuations. Centimeter-level reductions in RMSE indicate that the predicted groundwater levels are, on average, closer to the observations and can improve the accuracy with which the magnitude and timing of groundwater-level changes are represented. In particular, in areas subject to intensive agricultural pumping, more accurate identification of rapid groundwater-level decline and recovery can improve the resolution of groundwater dynamics monitoring, anomaly detection, and groundwater-level early warning in overexploited areas, while also providing a more reliable predictive basis for subsequent adjustment of pumping intensity and zonal groundwater management. Therefore, the significance of the proposed improvements lies not only in the reduction of statistical errors, but also in the more detailed quantitative representation of actual groundwater-level dynamics achieved by incorporating pumping disturbances and aquifer-specific response differences into the prediction framework.

Guantao County, a typical agricultural groundwater-extraction area in the North China Plain, was selected as the study area. Based on daily groundwater-level, meteorological, and pumping data from four shallow and four deep monitoring wells, a process-guided GRU model for multi-timescale groundwater-level prediction was developed to account for pumping disturbances and aquifer-specific response differences. The model was designed to incorporate simplified hydrological process knowledge into data-driven prediction through soft process guidance and to examine the role of pumping information in groundwater-level prediction across different aquifer types and temporal scales. Given the limited size of the monitoring network used in this study, future work could incorporate additional monitoring wells, the spatial distribution of pumping wells, aquifer structure, and more detailed pumping data to further improve the spatial generalizability of the model and its ability to represent groundwater responses to pumping disturbances. Overall, the main findings can be summarized as follows:

(1) A PG-GRU groundwater-level prediction framework was developed to account for the differentiated responses of shallow and deep wells. Building on the data-driven predictive capability of the GRU, knowledge of groundwater-level changes, meteorological recharge and depletion, agricultural pumping disturbances, and groundwater-level recovery was incorporated into model training in the form of lumped process residuals, with separate process-guidance relationships formulated for shallow and deep wells. This framework does not solve the complete governing equations of groundwater flow; rather, available hydrological forcing information is used to impose soft process guidance on GRU training. It therefore provides a modeling approach that combines data-driven capability with process-based prior knowledge for groundwater-level prediction under agricultural pumping disturbances.
(2) The PG-GRU generally achieved lower group-mean prediction errors than the comparison models at both the daily and dekadal (10-day) scales. Compared with MLR, SVR, and the conventional GRU, the PG-GRU achieved the lowest group-mean RMSE for both shallow and deep wells at both temporal scales. Relative to the conventional GRU, the mean RMSE values for the shallow and deep wells at the daily scale decreased from 0.1967 m to 0.1632 m–0.1825 m and 0.1533 m, corresponding to relative reductions of approximately 7.2% and 6.1%, respectively. At the dekadal scale, the corresponding mean RMSE values decreased from 1.0307 m to 1.7120 m–0.9890 m to 1.6756 m, representing reductions of approximately 4.0% and 2.1%, respectively. However, the magnitude of improvement was not fully consistent across monitoring wells or evaluation metrics, with particularly pronounced well-specific differences observed for the shallow wells at the dekadal scale. Therefore, the performance gains of the PG-GRU should be interpreted as an overall incremental improvement rather than a consistent advantage across all monitoring wells and evaluation metrics.
(3) The contribution of agricultural pumping information to model performance was dependent on both aquifer type and temporal window. For the shallow wells, explicitly incorporating daily pumping as a process-guidance term generally helped reduce prediction errors. For the deep wells, however, the contribution of pumping information depended more strongly on the appropriate representation of the cumulative response window. Sensitivity analysis using 30-, 60-, and 90-day moving-average pumping series showed that the preferred temporal window varied among the deep wells. The 60-day window provided relatively stable overall performance as the unified main-model configuration, whereas the 30- and 90-day windows yielded lower RMSE values for some individual wells. These results indicate that deep groundwater responses to agricultural pumping disturbances are not governed by a single fixed accumulation timescale. Therefore, in groundwater-level prediction for agricultural pumping areas, pumping disturbances should not only be incorporated into the model but also be represented appropriately according to aquifer type and well-specific response timescales.

### Limitations of the study

However, several limitations of the proposed approach should be acknowledged. First, the process residual formulated in this study provides lumped soft guidance rather than directly solving the complete partial differential equations governing groundwater flow. Therefore, interpret the model parameters as effective lumped response weights optimized in the normalized model space, rather than as actual hydrogeological parameters. For the deep wells, vertical leakage and regional lateral groundwater flow were not explicitly represented in the process residual. Their integrated effects may have been partially captured by historical groundwater levels and the data-driven component of the GRU, or reflected in the effective response weights. This limits both the physical interpretability of these weights and the transferability of the model to settings with different boundary and recharge conditions. Second, the model was validated using four shallow and four deep monitoring wells, so the monitoring network remains limited. Therefore, the observed differences between the shallow- and deep-well groups should not be interpreted as universally applicable intrinsic relationships between unconfined and confined aquifers. Uncertainty also remains when extrapolating the findings to areas without monitoring wells, and the conclusions require further validation using additional monitoring wells under different pumping intensities and hydrogeological conditions.

In addition, although the pumping data represent regional agricultural groundwater abstraction disturbances, uncertainties remain in the reconstructed groundwater-abstraction series. For the period 2017–2022, the dataset used in this study did not include the original electricity-consumption records, well-specific electricity-to-water conversion coefficients, or calibration-error information. Therefore, the uncertainty associated with the reconstructed groundwater-abstraction volumes could not be quantitatively assessed using the available information. Such uncertainty may propagate to the optimized pumping-response weight *η*, the comparison among different cumulative pumping windows, and groundwater-level predictions. Uncertainties also remain in the spatial distribution of pumping, the actual pumping intensity near individual monitoring wells, irrigation return flow, and surface-water recharge processes. These uncertainties may affect the model’s ability to reproduce local groundwater-level variations and may be particularly relevant at wells with relatively large prediction errors. For the deep wells, 30-, 60-, and 90-day moving-average pumping series were used to represent cumulative pumping disturbances. Although these predefined windows provided an initial representation of differences in pumping-response timescales, the window lengths remained prescribed rather than being adaptively identified by the model.

Future research could be extended in several directions. First, additional groundwater monitoring wells, the spatial distribution of pumping wells, aquifer architecture, and stratigraphic parameters could be incorporated to better represent spatial heterogeneity and local hydrogeological conditions. Second, more detailed information on groundwater abstraction, irrigation practices, surface-water recharge, and managed aquifer recharge could be introduced to improve the representation of agricultural pumping disturbances. Third, cross-well transfer, multi-well joint modeling, or graph-structured process-guided models could be explored to further improve model generalizability across regional groundwater systems. Fourth, learnable response timescales for pumping disturbances could be developed so that more appropriate cumulative pumping windows can be identified automatically according to the dynamic characteristics of individual monitoring wells.

## Resource availability

### Lead contact

Requests for further information and resources should be directed to and will be fulfilled by the lead contact, Baizhong Yan (jluybz@126.com).

### Materials availability

This study did not generate new unique materials.

### Data and code availability

• Meteorological data used in this study were derived from the ChinaMet dataset provided by the National Cryosphere Desert Data Center. The processed meteorological dataset used in this study has been deposited in Zenodo and is publicly available at https://doi.org/10.5281/zenodo.22897645.
• Groundwater-level and agricultural groundwater-pumping records used in this study are subject to data-provider and government data-management restrictions and cannot be deposited in a public repository. Requests for access should be directed to the lead contact and will be handled subject to approval by the relevant data holders.
• All original code used for the GRU, PG-GRU, PG-GRU-noQ, MLR, and SVR analyses is provided as Data S1 in the supplemental information accompanying this article.
• Any additional information required to reanalyze the data reported in this paper is available from the lead contact upon request.

## Acknowledgments

This work was financially supported by the 10.13039/501100003482Hebei Provincial Department of Education Key Cultivation Special Project for Basic Research (JCZX2026038), the 10.13039/501100003482Hebei Provincial Department of Education Funding Project for Cultivating Innovative Ability of In-Service Postgraduates (CXZZDD202622), the General Program of the 10.13039/501100003787Natural Science Foundation of Hebei Province (D2024403005), and the Hebei Key Laboratory of Mountain Geological Environment (no HBKLMGE202402).

## Author contributions

X.X.: writing - original draft, methodology, software, formal analysis, investigation, and visualization. B.Y.: writing - review, editing, conceptualization, supervision, funding acquisition, and project administration. T.W.: writing – review and editing, supervision, methodology, and conceptualization. C.H.: visualization, validation, and data curation. C.W.: validation, investigation, and data curation. Y.Z.: visualization, validation, and data curation. J.Lv: visualization, validation, and data curation. M.Z.: visualization, validation, and data curation.

## Declaration of interests

The authors declare that they have no known competing financial interests or personal relationships that could have appeared to influence the work reported in this paper.

## STAR★Methods

### Key resources table

**Table:** 

REAGENT or RESOURCE	SOURCE	IDENTIFIER
**Deposited data**		

Meteorological dataset	National Cryosphere Desert Data Center	Zenodo: https://doi.org/10.5281/zenodo.22897645
Groundwater-level and pumping data	Data provider	Restricted access/N/A

**Software and algorithms**		

Python	Python Software Foundation	Version 3.13.5
PyTorch	PyTorch	Version 2.7.0
GRU, PG-GRU, PG-GRU-noQ, MLR, and SVR source code	This paper	Data S1

### Method details

#### Study area

The study area is located in Guantao County, Handan City, Hebei Province, in the southern North China Plain, China (Figure 4). Guantao County is a typical agricultural area of the North China Plain, characterized by generally flat terrain, extensive cultivated land, and high demand for water resources to support agricultural production. Influenced by the monsoon climate, precipitation in the study area is unevenly distributed within the year and is mainly concentrated in summer. In contrast, the winter wheat–summer maize cropping system requires substantial irrigation water, making groundwater a long-term and important source for regional agricultural irrigation. The North China Plain, where Guantao County is located, is one of the typical groundwater-overexploited regions in China. Groundwater overexploitation in the North China Plain has long been associated with increasing agricultural irrigation demand, uneven spatiotemporal distribution of surface water resources, and continuous groundwater abstraction.

Guantao County is characterized by generally flat terrain, with a gentle overall slope from southwest to northeast. Ground surface elevation decreases from approximately 43 m in the south to approximately 36 m in the north, representing a typical alluvial plain landform. Influenced by deposition and erosion associated with ancient river channels, microgeomorphic units such as abandoned channels, gentle ridges, and depressions are developed in the area. Quaternary unconsolidated sediments are widely distributed across the study area, and the aquifer media are mainly composed of sand, silty sand, clay, and silty clay. Sand layers and clayey layers are interbedded, forming a hydrogeological structure in which multiple aquifers and relatively aquitard layers occur alternately. This sedimentary structure results in a certain degree of spatial heterogeneity in groundwater occurrence and flow conditions, and provides the geological basis for different dynamic responses between shallow and deep groundwater.^34^

As shown in Figure 4C, groundwater abstraction conditions in Guantao County exhibit clear spatial heterogeneity and can be divided into three zones: a shallow groundwater abstraction zone, a combined shallow–deep groundwater abstraction zone, and a deep groundwater abstraction zone. This zoning reflects differences in the dominant groundwater development conditions across the study area. The eight monitoring wells selected in this study are distributed across all three zones: S4 is located in the shallow groundwater abstraction zone; S1, S2, S3, and D4 are located in the combined shallow–deep groundwater abstraction zone; and D1, D2, and D3 are located in the deep groundwater abstraction zone. Therefore, the monitoring network covers the major groundwater abstraction zones in Guantao County rather than being concentrated within a single development setting, thereby providing a spatially differentiated basis for evaluating groundwater-level dynamics in shallow and deep monitoring wells.

#### Data processing

Daily groundwater-level data from four shallow and four deep monitoring wells in Guantao County were selected as the prediction targets (Figure 5), with the corresponding data periods summarized in Table 1. Considering that groundwater-level variations in the study area are jointly influenced by meteorological conditions and agricultural pumping activities, precipitation, potential evapotranspiration (PET), and groundwater abstraction were selected as the primary driving variables. Precipitation and PET data were obtained from the National Cryosphere Desert Data Center at a spatial resolution of 0.01°.^35^^,^^36^ Daily agricultural groundwater abstraction data from 2017 to 2024 were used. For 2017–2022, daily abstraction volumes were converted from measured daily electricity-consumption records of agricultural pumping wells using a locally calibrated electricity-to-water conversion relationship. The conversion relationship was established and validated using pump-operation records from representative villages and households together with field-survey data. For 2023–2024, daily abstraction volumes were obtained from area-wide metering data for the study area.

For the representation of pumping disturbances, daily groundwater abstraction Q was used in the shallow-well model to characterize short-term agricultural pumping disturbances. Because deep groundwater levels may exhibit delayed and cumulative responses to pumping, 30-, 60-, and 90-day moving-average abstraction series were further constructed from the daily Q data for the deep-well model to represent pumping disturbances over different cumulative time windows.

Before model development, temporal consistency checks and missing-value processing were performed for groundwater levels and all driving variables, and supervised learning samples were then constructed in chronological order. The dataset was divided chronologically into training, validation, and test sets for model training, hyperparameter optimization, and final performance evaluation, respectively. All input variables and prediction targets were normalized using Min–Max scaling. The normalization parameters were calculated exclusively from the training set and subsequently applied to the validation and test sets to prevent information leakage from future observations. Model predictions were inverse-transformed to the original scale before the evaluation metrics were calculated and the results were analyzed.

#### Aquifer-differentiated process-guided GRU framework

Groundwater-level variations across the eight monitoring wells in the study area exhibited both similarities driven by common external forcings and differences associated with individual wells and aquifer responses. This indicates that, under the combined influence of agricultural pumping disturbances and regional meteorological conditions, groundwater-level dynamics in shallow and deep wells are not completely independent, but neither can their response processes be regarded as homogeneous. Therefore, a conventional data-driven model that relies solely on a unified statistical mapping may be insufficient to fully represent the differentiated responses of different aquifer types to meteorological conditions and pumping disturbances.

As shown in Figure 6, a GRU was adopted as the backbone for groundwater-level time-series prediction, and an aquifer-differentiated process-guided GRU model was developed. Unlike the conventional GRU, in which network parameters are updated solely by minimizing the data-fitting error, the proposed model incorporates a process-residual term based on groundwater-level dynamics into the loss function. Thus, while fitting the observed groundwater levels, model training is also guided by precipitation recharge, potential evapotranspiration losses, pumping disturbances, and groundwater-level recovery processes.

Considering the differences in groundwater responses between shallow and deep aquifers, separate process residuals were formulated for the shallow and deep wells. For the shallow wells, groundwater-level variations are more closely related to near-surface hydrological processes; therefore, precipitation recharge, potential evapotranspiration losses, daily groundwater abstraction, and groundwater-level recovery were jointly considered in the process guidance. For the deep wells, the direct effects of daily precipitation and potential evapotranspiration on groundwater-level variations are relatively weak, whereas groundwater-level changes are more strongly associated with sustained pumping pressure and its delayed cumulative effects. Accordingly, moving-average groundwater abstraction and groundwater-level recovery were emphasized in the process guidance.

It should be noted that the process guidance in the model was primarily constructed from groundwater-level continuity and available driving variables, including precipitation, potential evapotranspiration, and groundwater abstraction. The associated parameters should therefore be interpreted as effective lumped response coefficients under normalized conditions.

#### Shallow-well process guidance

Shallow groundwater is closely connected to near-surface hydrological processes, and its level variations are typically influenced by the combined effects of precipitation infiltration recharge, potential evapotranspiration losses, agricultural pumping disturbances, and groundwater-system recovery. Based on this understanding, a lumped process-guidance relationship incorporating recharge, depletion, pumping, and recovery terms was formulated for the shallow wells.

Groundwater-level variations in the shallow wells can be conceptualized as:

(Equation 1) $$w_{\Delta h}\frac{dh}{dt}=\alpha P-\gamma PET-\eta Q-\beta \left(h-h_{ref}\right)$$

Where *h* is the groundwater level; $\frac{dh}{dt}$ is the rate of groundwater-level change; *P* is precipitation; *PET* is potential evapotranspiration; *Q* is daily groundwater abstraction; *h*_*ref*_ is the reference groundwater level, which was defined as the mean groundwater level during the training period and used in the process-residual calculation in the normalized model space. *w*_Δ*h*_, *α*, *γ*, *η* and *β* are effective lumped response coefficients. Specifically, *αP* represents the effect of precipitation recharge on shallow groundwater-level rise; *γPET* represents water loss due to potential evapotranspiration; *ηQ* represents groundwater-level decline induced by agricultural pumping; and *β*(*h*-*h*_*ref*_) represents the recovery of groundwater levels toward the reference state.

Under daily-scale discretization, the rate of groundwater-level change can be approximated as:

(Equation 2) $$\frac{dh}{dt}\approx h_{t}^{pred}-h_{t-1}$$

where $h_{t}^{pred}$ is the groundwater level predicted by the model at the current time step, and *h*_*t*-1_ is the groundwater level at the previous time step. Accordingly, the process residual for the shallow wells can be written as:

(Equation 3) $$R_{S}=w_{\Delta h}\left(h_{t}^{pred}-h_{t-1}\right)-\alpha P_{t}+\gamma {PET}_{t}+\eta Q_{t}+\beta \left(h_{t}^{pred}-h_{ref}\right)$$

where *R*_*S*_ denotes the process residual for the shallow wells, and *P*_*t*_, *PET*_*t*_ and *Q*_*t*_ denote precipitation, potential evapotranspiration, and daily groundwater abstraction at time *t*, respectively. Minimizing *R*_*S*_ during model training promotes consistency between shallow groundwater-level changes and the lumped relationship comprising precipitation recharge, potential evapotranspiration loss, agricultural pumping disturbance, and groundwater-level recovery.

#### Deep-well process guidance

Compared with shallow groundwater, deep groundwater is less directly affected by daily precipitation and potential evapotranspiration, and its level variations more strongly reflect sustained pumping pressure and delayed cumulative responses. For deep wells, changes in daily pumping alone may not adequately represent the pumping pressure experienced by the deep groundwater system. Therefore, moving-average pumping series were used to represent cumulative pumping disturbances over different temporal windows, and a process residual dominated by cumulative pumping and groundwater-level recovery terms was formulated for the deep wells.

Groundwater-level variations in the deep wells can be conceptualized as:

(Equation 4) $$w_{\Delta h}\frac{dh}{dt}=-\eta Q_{w}-\beta \left(h-h_{ref}\right)$$

where *Q*_*w*_ is the moving-average groundwater abstraction series; *w* is the moving-window length; and *w*_Δ*h*_, *η* and *β* are effective lumped response coefficients. In this relationship, -*ηQ*_*w*_ represents the effect of cumulative pumping disturbances on deep groundwater-level decline, whereas -*β*(*h*-*h*_*ref*_) represents the recovery of deep groundwater levels toward the reference state.

Under daily-scale discretization, the process residual for the deep wells can be written as:

(Equation 5) $$R_{d}=w_{\Delta h}\left(h_{t}^{pred}-h_{t-1}\right)+\eta Q_{w,t}+\beta \left(h_{t}^{pred}-h_{ref}\right)$$

where *R*_*d*_ denotes the process residual for the deep wells, and *Q*_*w*,*t*_ is the moving-average groundwater abstraction at time *t* calculated using a window length of *w*. In this study, the window length was set to *w* = 30, 60, 90 days to represent short-, medium-, and longer-term cumulative pumping disturbances and to examine differences in deep groundwater-level responses across cumulative pumping windows.

Notably, precipitation and potential evapotranspiration were not directly included in the deep-well process residual. This does not imply that deep groundwater-level variations are entirely independent of regional meteorological conditions. Rather, the direct flux effects of daily precipitation and potential evapotranspiration on deep groundwater levels are generally weak and are difficult to represent as instantaneous recharge or depletion terms in a daily-scale residual. Therefore, in the deep-well model, precipitation and potential evapotranspiration were retained as GRU input variables for data-driven learning to represent the regional meteorological background and its potential indirect effects, whereas the process residual focused on cumulative pumping disturbances and groundwater-level recovery.

#### Loss function and model training

Model training was jointly governed by the data-fitting loss and the process-guidance loss. For any sample, the data residual can be expressed as:

(Equation 6) $$R_{data}=h_{t}^{pred}-h_{t}^{obs}$$

where $h_{t}^{pred}$ is the groundwater level predicted by the model at the current time step, and $h_{t}^{obs}$ is the corresponding observed groundwater level. The data-fitting loss was defined using the mean squared error:

(Equation 7) $$L_{data}=\frac{1}{N}\sum_{i=1}^{N}{\left(h_{i}^{pred}-h_{i}^{obs}\right)}^{2}$$

For the shallow wells, the process-guidance loss was defined as:

(Equation 8) $$L_{pg}^{s}=\frac{1}{N}\sum_{i=1}^{N}{\left(R_{s,i}\right)}^{2}$$

For the deep wells, the process-guidance loss was defined as:

(Equation 9) $$L_{pg}^{d}=\frac{1}{N}\sum_{i=1}^{N}{\left(R_{d,i}\right)}^{2}$$

where *R*_*s*,*i*_ and *R*_*d*,*i*_ are the shallow- and deep-well process residuals, respectively, for the *i* sample. The total loss function can therefore be expressed as:

(Equation 10) $$L_{total}=\lambda_{data}L_{data}+\lambda_{pg}L_{pg}$$

where *λ*_*data*_ and *λ*_*pg*_ are the weighting coefficients for the data-fitting loss and process-guidance loss, respectively, and are used to balance the accuracy of fitting the observations and the strength of process-consistency guidance. In this study, the data-loss weight *λ*_*data*_ was fixed at 1, whereas the process-guidance-loss weight *λ*_*pg*_ was optimized to avoid process guidance that was either too weak to be effective or too strong to impair the model fit to the observations.^37^^,^^38^^,^^39^^,^^40^

During model training, the GRU network parameters and the effective lumped response coefficients in the process-guidance equations were optimized simultaneously. For the shallow-well model, the effective lumped parameters included *w*_Δ*h*_, *α*, *γ*, *η* and *β*, corresponding to the groundwater-level change, precipitation recharge, potential evapotranspiration loss, pumping disturbance, and groundwater-level recovery terms, respectively. For the deep-well model, the effective lumped parameters included *w*_Δ*h*_, *η* and *β*, representing deep groundwater-level change, cumulative pumping disturbance, and groundwater-level recovery, respectively.

### Quantification and statistical analysis

For group-level performance comparisons, *n* = 4 monitoring wells for each aquifer group (four shallow wells and four deep wells, respectively). Group-level performance metrics are reported as the mean and standard deviation (SD) across the four monitoring wells within each group. Model-training stability was additionally evaluated using five random seeds (42, 52, 62, 72, and 82). No inferential statistical tests were performed. The numbers of daily observations available for each monitoring well are reported in Table 1.

#### Model implementation and training setup

To ensure temporal independence among model training and evaluation subsets, the data were divided chronologically into training, validation, and test sets at proportions of 70%, 15%, and 15%, respectively. The models were trained using the AdamW optimizer, and multiple random seeds and gradient clipping were employed to improve training stability. Network parameters, including hidden-layer size, number of network layers, and the weight assigned to the process-residual loss, together with the associated effective lumped response coefficients, were optimized using Optuna-based Bayesian optimization.^41^ The corresponding parameter search ranges are summarized in Table 2. In addition, a 60-day moving-average pumping series, Q, was used by default in the main model to represent cumulative pumping disturbances for the deep wells. The optimized process coefficients are provided in Table S1.

All model computations were performed under Windows 11. The models were implemented in Python 3.13.5 using PyTorch 2.7.0.^42^^,^^43^ CUDA 13.0 was enabled for GPU acceleration during model training. The computational platform was equipped with an NVIDIA GeForce RTX 5060 Laptop GPU, an Intel processor, and 16 GB of memory.

#### Model performance evaluation

To quantitatively evaluate the groundwater-level prediction performance of different models, root-mean-square error (RMSE), Nash–Sutcliffe efficiency (NSE), Kling–Gupta efficiency (KGE), and bias were used as evaluation metrics in this study. RMSE was used to measure the average magnitude of the error between predicted and observed values, with smaller values indicating lower prediction errors. NSE was used to evaluate the ability of the predicted series to reproduce the variations in the observed series; values closer to 1 indicate better simulation of groundwater-level dynamics. KGE was used to assess the overall ability of the model to simulate groundwater-level variations. A KGE value closer to 1 indicates better agreement between the predicted and observed series in terms of correlation, variability, and mean bias. Bias was used to quantify the systematic deviation of model predictions. A bias value closer to 0 indicates smaller systematic deviation; positive values indicate overall overestimation of groundwater levels, whereas negative values indicate overall underestimation.

(Equation 11) $$RMSE=\sqrt{\frac{1}{N}\sum_{i=1}^{N}{\left(h_{i}^{pred}-h_{i}^{obs}\right)}^{2}}$$

(Equation 12) $$NSE=1-\frac{\sum_{i=1}^{N}{\left(h_{i}^{obs}-h_{i}^{pred}\right)}^{2}}{\sum_{i=1}^{N}{\left(h_{i}^{obs}-{\bar{h}}^{obs}\right)}^{2}}$$

(Equation 13) $$KGE=1-\sqrt{{\left(r-1\right)}^{2}+{\left(\alpha -1\right)}^{2}+{\left(\beta -1\right)}^{2}}\;\left(\alpha =\frac{\sigma_{pred}}{\sigma_{obs}},\beta =\frac{\mu_{pred}}{\mu_{obs}}\right)$$

(Equation 14) $$Bias=\frac{1}{N}\sum_{i=1}^{N}\left(h_{i}^{pred}-h_{i}^{obs}\right)$$

where $h_{i}^{pred}$ and $h_{i}^{obs}$ denote the predicted and observed groundwater levels of the *i*-th sample, respectively; ${\bar{h}}^{obs}$ is the mean observed groundwater level; *r*is the correlation coefficient between the predicted and observed values; *α* is the ratio of the standard deviation of the predicted values to that of the observed values; and *β* is the ratio of the mean of the predicted values to that of the observed values. *σ*_*pred*_ and *σ*_*obs*_ denote the standard deviations of the predicted and observed values, respectively, and *μ*_*pred*_ and *μ*_*obs*_ denote the means of the predicted and observed values, respectively.

## Supplemental information

Table S1. Optimized process-guidance coefficients for the eight monitoring wells at daily and dekadal (10-day) scales, related to STAR Methods

Table S2. Performance metrics of GRU, PG-GRU, and PG-GRU-noQ across five random seeds at daily and dekadal (10-day) scales, related to STAR Methods

Data S1. Source code for GRU, PG-GRU, PG-GRU-noQ, MLR, and SVR analyses, related to STAR Methods
